# Rapid stool antigenic test for typhoid fever among suspected cases, Northeast, Ethiopia

**DOI:** 10.1038/s41598-023-27909-5

**Published:** 2023-01-12

**Authors:** Alene Geteneh, Selamyhun Tadesse, Sirak Biset, Lencho Girma, Paulos Fissiha

**Affiliations:** 1 Department of Medical Laboratory Science, College of Health Sciences, Woldia University, Woldia, Ethiopia; 2 Department of Medical Microbiology, School of Biomedical and Laboratory Sciences, University of Gondar, Gondar, Ethiopia; 3 Department of Medical Laboratory Science, Mizan Aman College of Health Sciences, Aman, Ethiopia; 4 Felege Hiwot Referral Hospital, Bahir Dar, Ethiopia

**Keywords:** Medical research, Diseases, Infectious diseases, Microbiology, Infectious-disease diagnostics

## Abstract

Typhoid fever continued to be the key cause of morbidity and mortality in developing countries with poor hygienic practices and limited access to safe drinking water. The Widal card agglutination test is the main diagnostic tool in Ethiopia, which is limited in differentiating the overlapping symptoms with other acute febrile illnesses such as malaria and viral enteritis. This eventually leds to unnecessary antibiotic use and eventual drug resistance. Therefore this study wants to assess the burden and associated potential risk factors of typhoid fever among suspected cases using the typhoid rapid stool antigen test in Northeast Ethiopia. A hospital-based cross-sectional study was conducted at Gaint and Meket Shediho primary hospitals from May to July 2021. A total of 255 patients clinically suspected of typhoid fever, and willing to grant informed consent were included systematically. The demographic and hygiene-related variables were collected using a pre-tested structured questionnaire. The rapid stool antigenic test and xylose-lysine-deoxycholate agar (XLD) stool culture were evaluated for the level of agreement. The present study indicated that the prevalence of typhoid fever was 15.3%. This displayed that the human-restricted infectious disease, typhoid fever remained a challenge to Ethiopians. Washing hands with soap, history of typhoid fever, having previous history of hospitalization, and chronic underlying disease was the significant potential factor for typhoid fever. The higher agreement of the rapid stool antigenic test with the stool culture can indicate the factual burden of typhoid fever in the suspected population. This could minimize empiric treatment and the possible emergence of drug resistance. Thus, resource-poor settings may need to look for a rapid and reliable stool antigenic test.

## Introduction

The 2019 global burden report indicated that typhoid (caused by *Salmonella typhi*) and paratyphoid fever (caused by *Salmonella paratyphi*) was the 11th–14th top leading causes of morbidity and mortality worldwide since 1990^1^. Patients aged 0–9 years were shown to be more vulnerable than those aged 10–24 years^1,2^. Developing countries contributed to more than 87% (nearly a 12.5 milion) of the global cases^3^. This could be due to the higher HIV infection, lack of *Salmonella typhi* vaccination, lack of sanitations, exposure to unhygienic and polluted environment, and inaccessibility of safe drinking water in developing nations^2–6^. Age of patients, history of enteric fever infections, rural residency, use of well and spring water for drinking, consumption of raw milk, level of education and consumption of raw meat were identified as the predictors of typhoid fever to Ethiopians^7–9^. Investigating symptomatic cases for sanitary conditions of environment they live, accessibility of safe drinking water sources, and hygienic practices is imperative for devising contextual solutions to the majority of population, where these symptomatic cases have drawn.

There were no population-based epidemiologic data for typhoid fever in Ethiopia^10,11^. However, few health facility-based cross-sectional studies indicate that prevalence of typhoid fever was up to 20%^12–14^. Although the gold standard diagnosis of typhoid fever is the isolation of bacterium from blood, bone marrow aspirates, urine, and rose spots (small red spots on the abdomen and chest)^15,16^; diagnosis of typhoid fever in Ethiopia merely depends on Widal card agglutinin test. This is due to the poor laboratory infrastructures, and the cost unaffordability of culture-based diagnosis in Ethiopia^13,17^.

The Widal test is limited to differentiate the overlapping symptoms from other acute febrile illnesses such as malaria and viral enteritis^6,18,19^. The false positivity of the Widal test for febrile illness would complicate and worsen the malarial and viral febrile illness^20,21^. The Widal test-based studies displayed exaggerated findings of 32.6%^13^, 68.4%^12^, 68.5%^17^, and 81%^22^ indicating the test unreliability. The non-specificity of the Widal test makes the control of typhoid fever more challenging and also led to the unnecessary use of antibiotics and eventual resistance^2,10,23^. Thus, a relatively simple, one-step procedure, sensitive, and reliable point-of-care test like a rapid stool antigen test should be in place to diagnose typhoid fever^24,25^.

The rapid antigenic tests evaluation on the different rapid diagnostic tests (RDTs) indicating the good diagnostic accuracy relative to the reference standard test of blood or bone marrow culture^24^. A comparison of diagnostic accuracy for salmonella species have been performed on two rapid kits; the SD Bioline One Step *Salmonella* Typhi Ag Rapid Detection Kit (Standard Diagnostics, Republic of Korea), and the *Salmonella* Ag Rapid Test (Creative Diagnostics, USA)^26^. Referring the salmonella positive blood cultures, the Creative Diagnostics kits were sensitive (78.3%) and specific (91%)^26^. The potential limitation of Creative Diagnostics noted was the high false negativity for the non- *Salmonella typhi* species such as *Salmonella Paratyphi* A and *Salmonella choleraesuis*^26,27^. Our study is interested to assess the burden and associated potential risk factors of typhoid fever among suspected cases using a typhoid rapid stool antigen test in Northeast Ethiopia.

## Results

### Prevalence of typhoid fever

A total of 255 typhoid suspected participants have enrolled in this study. The mean age of study participants was 35.5 years (± SD 9.82). The majority (56.1%) were females and rural residents (62.4%). The overall prevalence of typhoid fever was 15.3%. The majority of confirmed cases were from rural residents (84.6%), who had four and more family members (66.7%), who had no formal education (64.1%), who consumed stream water (87.2%), who had no hand washing basin (97.4%), with the habit of eating undercooked vegetables (92.3%), with history of typhoid fever (94.9%) and who had chronic illnesses (87.2%) relative to their counterparts, respectively (Table 2).

### Sensitivity, specificity, and predictive values of stool antigenic test

To nearly half of the randomly selected study participants; both culture and rapid stool antigenic tests were carried out as a pilot. The two methods were shown to agree by 86.7% with a kappa value of 0.659, and the level of agreement was statistically significant (P < 0.001). Similarly, the sensitivity, specificity, and predictive values of stool antigenic test in diagnosing typhoid fever were estimated considering stool culture as a standard method.

Accordingly, the sensitivity, specificity, positive predictive (PPV), and negative predictive (NPV) values were found 75%, 91%, 75%, and 91% respectively in the present study (Table 1). Thus, the sensitivity and specificity indicated the concordance of the stool antigenic test to the XLD stool culture.

**Table 1:** Pilot depicting sensitivity, specificity, and predictive values of stool antigenic test against XLD stool culture, Northeast, Ethiopia, 2021.

	XLD stool culture		Total	
	Positive	Negative		
Stool antigenic test				
Positive	24	8	32	
Negative	8	80	88	
Total	32	88	120	

Sensitivity	Specificity	PPV	NPV	Kappa
Diagnostic test parameters				
75%	90.9%	75%	90.9%	0.659

### Socio-demographic and clinical factors of typhoid fever

The bivariate analysis has shown that residence, educational status, family size, sources of drinking water, washing hands before a meal, availability of latrine, ingestion of undercooked vegetables, history of typhoid fever, history of previous hospitalization, having chronic underlying diseases, and habit of trimming nail were statistically correlated with the prevalence of typhoid fever (p < 0.05). More than half (51.3%) of participants who do not wash their hands before meals developed typhoid fever (p < 0.005). However, the multivariable logistic regression indicated that washing with soap, history of typhoid fever, history of previous hospitalization, and history of chronic underlying disease was the only identified potential factors for typhoid fever (p < 0.05) in this study. Patients with a history of typhoid fever, a history of previous hospitalization, and a history of chronic underlying disease were 10.94, 2.83, and 6.22 times more likely to develop typhoid fever than their counterparts, respectively (Table 2).

**Table 2:** Bivariate and multivariable analysis of socio-demographic and clinical variables of typhoid fever suspected patients at Gaint and Meket Shediho primary hospitals, Ethiopia, 2021.

Characteristics (n = 255)	Typhoid fever positive, No (%)	Typhoid fever negative, No (%)	COR (95% CI)	P-value	AOR (95% CI)	P- value
Age (years)						
10–24	3 (7.7%)	35 (16.2%)	0.13 (0.02, 0.73)	0.02	0.48 (0.03, 8.05)	0.45
25–39	16 (41.0%)	107 (49.5%)	0.22 (0.06, 0.88)	0.032	0.27 (0.03, 2.06)	
40–54	16 (41.0%)	68 (31.5%)	0.35 (0.09, 1.40)	0.138	0.26 (0.04, 1.65)	
≥ 55	4 (10.3%)	6 (2.8%)	1			
Residence						
Rural	33 (84.6%)	126 (58.3%)	3.97 (1.58, 9.77)	0.003		
Urban	6 (15.4%)	90 (41.7%)	1			
Educational status						
No formal education	25 (64.1%)	86 (39.8%)	5.72 (1.65, 19.81)	0.006		0.56
Primary	11 (28.2%)	71 (32.9%)	3.05 (0.81, 11.43)	0.03		
Secondary and above	3 (7.7%)	59 (27.3%)	1			
Family size						
< 4	15 (33.3%)	136 (63%)	1			
≥ 4	24 (66.7%)	80 (37%)	2.72 (1.35, 5.49)	0.005		
Water source						
Stream	34 (87.2%)	12, 658.3%)	4.86 (1.83, 12.90)	0.001		
Tap	5 (12.8%)	90 (41.7%)	1			
Washing hands before meal						
Yes	19 (48.7%)	161 (74.5%)	1			
No	20 (51.3%)	53 (24.5%)	3.24 (1.61, 6.52)	0.001		
Handwashing basin						
Yes	1 (2.3%)	2712.5%)	1			
No	38 (97.4%)	189 (87.5%)	5.43 (0.72, 41.2)	0.102		
Washing with soap						
Yes	2 (5.1%)	3 (1.4%)	1			
No	37 (94.9%)	213 (98.6%)	0.26 (0.04, 1.61)	0.15	0.018 (0.0, 0.96)	0.048
Latrine availability						
Yes	32 (82.1%)	207 (95.8%)	5.03 (1.75, 14.56)	0.003		
No	7 (17.9%)	9 (4.2%)	1			
Habit of eating undercooked vegetables						
Yes	36 (92.3%)	175 (81%)	2.81 (0.83, 9.58)	0.098		
No	3 (7.7%)	41 (19%)	1			
History of typhoid fever						
Yes	37 (94.9%)	137 (63.4%)	10.67 (2.5, 45.5)	0.001	10.94 (2.3, 52.9)	0.003
No	2 (5.1%)	79 (36.6%)	1			
History of hospitalization						
Yes	25 (64.1%)	94 (43.5%)	2.32 (1.14, 4.70)	0.02	2.83 (1.09, 7.37)	0.033
No	14 (35.9%)	122 (56.5%)	1			
History of chronic underlying disease						
Yes	5 (12.8%)	9 (4.2%)	3.38 (1.07, 10.70)	0.038	6.22 (1.13, 34.18)	0.035
No	34 (87.2%)	207 (95.8%)	1			
Habit of trimming nail						
Yes	5 (12.8%)	77 (35.6%)	1			
No	34 (87.2%)	139 (64.4%)	3.77 (1.42, 10.03)	0.008	3.60 (0.56, 23.35)	0.179

*No* number, *COR* crude odds ratio, *AOR* adjusted odds ratio.

## Discussion

Typhoid fever is yet a key public health issue globally with varying burdens in geography, period, and season^7,28^. The global estimate of typhoid fever varies between 11·9 million and 27·1 million cases per year, and developing countries share the great majority of cases^29,30^. The current study depicts the fact that poor resource settings with limited access to safe drinking water and poor food handling practices would have a higher burden of typhoid fever as indicated by the 15.3% prevalence.

This finding is in the range of the late reports in Ethiopia 0.5%^14^ to 20%^12^. Our finding (15.3%) is in agreement with the previous culture-based finding (20%) in Ethiopia^12^, and with culture-confirmed febrile patients in Tanzania (10.1%)^22^ and Nigeria (14.1%)^31^; but lower than the report in India (72%)^28^. This study used the rapid stool antigenic test to detect typhoid fever; a human-restricted and highly adapted invasive systemic disease of adults and children that shows little association with immunosuppression, caused by S.typhi and S.paratyphi^32,33^. The negative suspected cases by this rapid stool antigenic test might be related to the nontyphoid Salmonellae (NTS) serotypes though they are less frequent^34^. In our pilot study, the rapid stool antigenic test and XLD agar-based stool culture showed a substantial level of agreement (k = 0.659) in contrast to the Widal test^9,12^.

Typhoid fever is also an important infectious disease for Ethiopians^10,12^. The descriptive data from Jimma displayed the ever-increasing burden from 2015 to 2019 with varying incidence over seasons^3^. Previously, different observational studies revealed that the prevalence of typhoid fever in Ethiopia varies from 0.5 to 20%^7–9,12,14,17,35,36^. Some of the studies pointed out that food handling practice and the hygienic condition is as poor as 60% in some rural settings of Ethiopia^4,37^. This, and the current political instability; might cause a relatively higher finding in this study. However, the possible sources of discrepancies could be the variations in the study setting (hospital-based vs. community-based)^35^, the poor sanitation and accessibility of safe water, and season of study^2,4,37^.

The absence of coordinated nationwide epidemiologic data^10^ makes African to fame and perceive outbreaks, and febrile illness as typhoid^38^. To better understand the real challenges, data from resource-constrained settings are needed^11^. Utmost the reliance of diagnosis on the Widal test would also give a hyperbolic burden^9,12,17,18,31^, and overtreatment of suspected cases^14,17,31,38^ which may led to ever-increasing drug resistance in the near future^12,17,21,24,31^.

Regarding the associated factors of typhoid fever; washing with soap, history of typhoid fever, history of previous hospitalization, and having a chronic underlying disease were identified as potential factors of typhoid fever (P < 0.05). After initial treatment, patients with typhoid fever may become an asymptomatic carriers of *Salmonella*, and continue to shade bacteria in their stool and urine, which can be a source of reinfection^39,40^ or reactivation of previous infections^7^. This could be the possible reason why the history of typhoid fever and previous history of hospitalization was found significantly associated with typhoid fever (P < 0.05) in the current study as in Bahir Dar, Ethiopia^7^. The significant association of having the chronic underlying disease with current typhoid fever might be explained by the fact that *Salmonella* infection was not cleared from the body due to the weakening immune system by the underlying chronic diseases^41,42^. Hand washing with soap was also identified as an associated factor; with odds of less than one indicative of its protective effect against typhoid fever. This hand-washing behavior was correlated with reducing the risk of typhoid in other studies^7,43^.

The comparative studies of culture and Widal test for typhoid fever in Ethiopia presented appreciable data: 0.5% vs.19%^14^, 4.1% vs. 32.6%^13^, 2.7% vs. 57.52%^9^, 20% vs. 68.4%^12^, 1.6% vs. 68.5%^17^, 10.1% vs. 81%^22^ and 3% vs. 33%^23^ respective prevalence of typhoid fever. According to previous studies, the Widal test has led to wrong treatment in 48–71%^17,22,31^ of febrile patients. Despite the poor agreement in the previous studies in Ethiopia^12,17^, Nigeria^31^, and Tanzania^22^, the rapid stool antigenic test has shown 86.7% agreement with the XLD agar culture result in this study. Thus, we recommended researchers and companies to evaluate this rapid stool antigenic test with the larger study population. This study was limited to detect *Salmonella paratyphi*, and *Salmonella typhi* below 10^4^/mL. Because of the hospital-based nature of the study, typhoid patients that don’t look for healthcare might be missed.

## Conclusions

The present study indicates typhoid fever remains a challenge to Ethiopia. We found that a history of typhoid fever, history of hospitalization, and chronic underlying disease was significant factor for typhoid fever. The higher agreement of the rapid stool antigenic test with the stool culture can indicate the factual burden of typhoid fever in the suspected population. This could minimize empiric treatment and the possible emergence of drug resistance. Thus, in resource-poor settings, the rapid and highly reliable stool antigenic test should be considered.

## Methods

### Study setup

A hospital-based cross-sectional study was conducted at Gaint and Meket Shediho primary hospitals from May to July 2021. These two primary hospitals are found in the Northern part of Amhara, serving millions of people in their catchment areas. Currently, these hospitals are using the rapid stool antigen test. However, the test results were not as the clinician expected (mostly negative) and were misperceived as false negatives due to the miss-customization of the Widal test, the old serological test.

### Eligibility criteria

A total of 255 patients clinically suspected of typhoid fever(patients with headache, abdominal pain, and fever (axillary temperature, ≥ 38 °C) for at least 3 days^8,42^), and willing to grant informed consent were included systematically, while suspected patients on antibiotics in the past 2 weeks were excluded during May to July 2021.

### Clinical and demographic data collection

Demographic characteristics such as age, sex, residence, and hygienic-related variables such as toilet availability, the habit of trimming nails, hand washing after toilet, water source, eating habit, previous history of hospitalization, history of chronic underlying disease, and history of typhoid fever were collected using a pre-tested structured questionnaire. After the interview a fresh stool specimens were collected using sterile screw-capped containers for rapid antigen testing.

### Laboratory procedure

The rapid *Salmonella typhi* card test (Creative Diagnostics, USA) is a qualitative immunoassay for the detection of *Salmonella* in faecal samples. During testing, the pre-coated anti-salmonella typhi antibodies in the membrane react with the *Salmonella typhi* antigen in the sample. Approximately 150 mg/mL of the faecal specimen from different parts was introduced into the buffer vial and shaken for good sample dispersion. The cap of the vial was broken off, and exactly 4 drops were dispensed into the specimen well. The result was read 10 min after dispensing the sample. The detection limit of this rapid test is 1 × 10^4^ *Salmonella typhi* /mL, and a too-small number of *Salmonella typhi* would test negative. Therefore, the test is a presumptive diagnosis of *Salmonella typhi* infections (typhoid fever).

The possible risk factors for typhoid fever were assessed using a pre-tested questionnaire. Pre-testing the questionnaire for at least 5% of the sample size is better to determine the potential effectiveness of the questionnaire in collecting the intended data. During the data collection, every questionnaire was checked for completeness. Both positive and negative controls were run at each batch (Creative Diagnostics, USA), the kits used were before their expiration date, and results were read within 10 min according to the manufacturer's instructions. Before the actual study, the level of agreement between the rapid stool antigenic test and stool culture was assessed as follows: fecal saline suspension was prepared for the formed stool specimen. While, the liquid stool was directly inoculated onto xylose-lysine-deoxycholate agar (XLD) (Oxoid, UK) at 35 °C for 24 h. The genus *Salmonella* was identified by the colony morphology, Gram staining, and biochemical test following standard bacteriological methods and this protocol is a modified version of Amsalu T^7^.

### Statistical analysis

Data were checked for completeness and entered into Epi-data v3.1. SPSS v25.0 was used for statistical analysis. Bivariate and multivariable logistic regression was done to identify factors associated with typhoid fever. Factors with a p-value of < 0.2 were considered substantial factors in the binary regression and included in the multivariable logistic regression. A p-value < 0.05 was considered statistically significant.

### Ethical approval and consent to participate

The study was approved by the Institutional Review Board of Woldia University. Written informed consent/assent was obtained from study participants, and parents or guardians of children, before including them in the study. In addition, all the information obtained from each study participant was coded to maintain confidentiality and positive results were communicated with responsible physicians for proper patient care. This research was carried out in line with the Helsinki declarations.

## Data Availability

All the relevant data are within the manuscript.
